# Molecular, Cytogenetic, and Hematological Analysis of Chronic Myeloid Leukemia Patients and Discovery of Two Novel Translocations

**DOI:** 10.1155/2021/4909012

**Published:** 2021-08-12

**Authors:** Muhammad Asif, Abrar Hussain, Abdul Wali, Nazeer Ahmed, Irfan Ali, Zafar Iqbal, Muhammad Amir, Muhammad Shafiq, Mahmood Rasool

**Affiliations:** ^1^Department of Biotechnology, BUITEMS, Quetta, Pakistan; ^2^Office of Research Innovation and Commercialization, BUITEMS, Quetta, Pakistan; ^3^Centre of Agricultural Biochemistry and Biotechnology, Agriculture University of Faisalabad, Pakistan; ^4^Clinical Laboratory Sciences Program, College of Applied Medical Sciences, King Saud Bin Abdulaziz University for Health Sciences/KAIMRC/SSBMT, National Guard Health Affairs, King Abdulaziz Medical City, Al-Ahsa, Saudi Arabia; ^5^Department of Biotechnology, University of Sialkot, Pakistan; ^6^Center of Excellence in Genomic Medicine Research, Faculty of Applied Medical Sciences, King Abdulaziz University, Jeddah, Saudi Arabia; ^7^Department of Medical Laboratory Technology, Faculty of Applied Medical Sciences, King Abdulaziz University, Jeddah, Saudi Arabia

## Abstract

Chronic myeloid leukemia (CML) is a disease of hematopoietic stem cells and is caused by the balanced translocations among the long arms of chromosomes 9 and 22, which are called the Philadelphia (Ph) chromosome. In this study, 131 CML patients were enrolled. Complete blood cell count was performed at the time of diagnosis for all the patients. Cytogenetic (karyotyping) examination using bone marrow samples was conducted on 76 CML patients for the confirmation of Ph-positive (9;22)(q34;q11) standard translocation, complex variant translocation, and additional chromosome abnormalities. FISH was performed on 38 patients for diagnostic purposes and on 39 patients for monitoring purposes. Twenty-two samples of CML patients were evaluated by reverse transcriptase PCR and real-time PCR for the patients who failed to respond against imatinib mesylate. In this study, 72 (54.96%) were males and 59 (45.03%) were females with a median age of 38.5 years. CBC values in the diagnosis process showed that 75 patients had high values of WBC being >100 × 10^3^/*μ*l, while 71 (58.01) patients exhibited reduced values of hemoglobin, i.e., <10.00 mg/dl, and high values of PLTs > 100 were observed in 40 (30.53%) patients. Cytogenetic results show that standard translocation was developed in 63 (82.89%), development of complex variant translocations in 4 (5.32%), additional chromosomal abnormalities (ACAs) in 3 (3.94%), and ACAs together with complex variant translocations in 1 (1.31%) patient. At the time of diagnosis, 61 (92.95%) patients were in the chronic phase, 4 (5.63%) were in the accelerated phase, and only 1 (1.40%) was in the blast crisis. Out of twenty-two patients, only 6 CML patients who were shifted from imatinib mesylate to nilotinib showed BCR-ABL-positive amplification. However, only 7 out of twenty-one patients exhibit BCR-ABL gene values ≥ 1 after three months of follow-up when analyzed by the quantitative real-time PCR. In conclusion, we found a novel five-way translocation 46XX,t(1;2;2;17;9;22)(p36.3,q21;q11.2,q21,q34,q11.2) and a novel four-way complex variant translocation 48XY,+8(8;17)(9;22),+der(22)(q11.2;q23)(q34;q11.2) in the accelerated phase.

## 1. Introduction

Chronic myeloid leukemia is a disease of hematopoietic stem cells. CML is a type of cancer caused by the balanced translocations between the long arms of chromosomes 9 and 22, t(9;22)(q13;q11), which are called the Philadelphia (Ph) chromosome and generally have the BCR-ABL gene fusion [1–3]. The formation of the BCR-ABL fusion gene takes place by transferring the 3 portions of the ABL from 9q34 to the 5 portions of the BCR gene on 22 q11.2 chromosomes. The appearance of the BCR-ABL chimeric protein increases the activity of protein kinase that plays the main part in the development of CML [4, 5]. CML is accountable for approximately 20% of all cases of leukemia with a prevalence of 1 to 1.5/100,000 [6, 7]. The median age of CML patients is from 45 to 55 years; however, all age groups, including children, are also affected. Moreover, 15-30% of CML cases are 60 years of age or older [8].

The translocation of chromosomes 9 and 22 is observed in about 90-95% of all CML cases [9, 10]; however, variant/complex translocation is established in about only 5-8% of CML cases [11–15]. The acquisition of additional cytogenetic aberrations is referred to as clonal evolution; in the Ph chromosome, positive cells have been thought to play a prime role in CML progression. The most common secondary cytogenetic abnormalities observed in CML patients with clonal evolution are trisomy 8, isochromosome 17, and duplicate Ph chromosome. And other cytogenetic aberrancies are trisomy 19, trisomy 21, trisomy 17, trisomy 22, and deletion of chromosomes such as -7 and -17 [14].

During diagnosis or clinical analysis, 50% of patients of CML are symptomless and are recognized by routine clinical tests [16]. Symptoms appear when the spleen becomes enlarged, WBC sufficiently raised to cause leukocytosis, anemia, hyperbasophilia progresses, or deviations of the platelet values that lead to thrombotic or hemorrhagic hitches [17]. The most important signs and symptoms of CML are skin lesions, yellowish color of the eyes and tiredness, occasional bleeding, weight loss, high fever, anemia, night sweats, abdominal pain, bruising spots on the entire body, bone pain, feeling exhausted, and burning ambiances below the feet [18].

Imatinib mesylate (IM) is a substantial oral therapy for the CML patients. It is an inhibitor of BCR-ABL protein tyrosine kinase and has the ability to induce a complete cytogenetic response in 65-90% CML-affected patients. The major function of this drug is that it blocks the proliferation and induces the apoptosis process of BCR-ABL expression in cells [15, 19–22]. However, a small percentage of CML patients show insensitivity to IM [23, 24]. There are many causes of resistance to IM in CML patients, in which the main cause is a disruption in treatment and reduced dosage. Resistance may also occur by additional cytogenetic aberrations or by the amplification of the BCR-ABL gene [25].

Nilotinib (Tasigna) is a second-generation oral therapy that inhibits tyrosine kinase activity with better effectiveness and selectivity for BCR-ABL than IM. Nilotinib was first permitted in the United States somewhere in 2007 for CML patients in CP and AP that showed resistance to IM. Nilotinib, similar to IM, binds to the kinase domain of ABL protein and displays a major role in the establishment of catalytically energetic conformation and finally inhibits the BCR-ABL signal transduction [26–28].

The current study was planned to investigate the CML patients for the detection of Ph-positive and negative cells (9;22)(34q;11q) and especially for the complex variant translocations, additional chromosomal abnormalities.

The current research is aimed at examining the molecular, cytogenetic, and hematological analysis of CML patients and investigating the effectiveness of current therapies, i.e., imatinib mesylate and nilotinib.

## 2. Materials and Methods

### 2.1. Study Design

The study was performed at Civil Hospital Quetta and Allied Hospital Faisalabad, Pakistan, between the periods of August 2014 and December 2018. Overall, 131 CML patients were enrolled in this study, in which 72 were males and 59 were females. A written informed consent form was filled by all persons who contributed to the current study. The study was approved by the local ethical committee of BUITEMS, Quetta, Pakistan.

### 2.2. Sampling and Data Collection

All the necessary data was collected by qualified medics and paramedical staff. For this purpose, a detailed questionnaire was designed which included name, age, sex, sign and symptoms, awareness about the disease, family history, duration of disease, and period of treatment. On the basis of age, patients were distributed into 4 groups, 16–30, 31–45, 46–60, and above 60 years, respectively.

### 2.3. Blood Collection for Complete Blood Cell Count Analysis

CBC was analyzed within 24-48 hours after the collection of blood using by a hematology analyzer. In CBC investigation, white blood cells (WBC), hemoglobin (Hbg), mean corpuscular hemoglobin concentration (MCHC), red blood cell distribution width (RDW), mean corpuscular volume (MCV), red blood cells (RBC), mean corpuscular hemoglobin (MCH), platelets, and number of blast cells were analyzed.

### 2.4. Cytogenetic Examination

The cytogenetic examination was done by bone marrow culture by a recognized technique. A maximum of 20 bone marrow metaphase cells were examined by GTG banded technique. Karyotyping was articulated by the International System for Human Cytogenetic Nomenclature [29].

### 2.5. Method of FISH Analysis

FISH was done by directly labeled dual-color LSI/CEP probes abiding by the probe dealer's directions (BCR-ABL-Oncor Ventana Medical Systems, Tucson, AZ, USA) for the finding of BCR-ABL. For this purpose, 200 or 500 metaphase/interphase cells were calculated for creating a BCR-ABL gene fusion rate [30, 31].

### 2.6. Amplification of BCR-ABL Fusion Oncogene

Venous blood of 3 ml was drawn from 22 CML subjects. The RNA was extracted by RNA method. The quantification of total RNA was performed using the NanoDrop™ 1000 Spectrophotometer (Eppendorf, USA) [32]. The cDNA was synthesized using the cDNA synthesis kit provided by Thermo Scientific (cat# 1621) as per manufacturer instruction [33, 34]. The cDNA was used as a template in the first round of the PCR while the amplified product of the first round was used as the template for the second round of the PCR. The sequences of the primer used in the nested PCR reaction were in round 1 B2A (F) 5′-TTCAGAAGCTTCTCCCTGACAT-3′ and ABL 4065 (R) 5′-CCTTCTCTAGCAGCTCATACACCTG-3′ and in round 2 BCR F4 (F) 5′-ACAGCATTCCGCTGACCATCAATA-3′ and U396 (R) 5′-GCCATAGGTAGCAATTTCCC-3 followed from Akram et al. [33].

### 2.7. Quantification of BCR-ABL Transcription by Quantitative Real-Time PCR

The total RNA of CML patients was isolated by the Trizol® reagent method. The cDNA was diluted and adjusted at 10 ng/*μ*l. The relative quantification of the BCR-ABL fusion region was amplified by using the primers B2A forwarded and ABL 4065 reverse. All the reactions were run in an iCycler thermal cycler using iQ5 multicolor real-time PCR machine (Bio-Rad). The reaction was performed by running all samples in triplicates on 96-well plates with necessary controls (negative, internal, and positive control). The GAPDH gene was used as an internal control. The thermal cycler conditions were 94°C for 10 min followed by 35 cycles of 94°C for 30 seconds (s), 54°C for 30 s, and 72°C for 30 s. A melt curve analysis (from 54 to 95°C with an increment of 0.5°C every 10 s) was conducted at the end of every run to verify the specificity of the amplified product. The reaction for relative quantification contained 2.5 *μ*l (25 ng) of template, 12.5 *μ*l of SYBR Green mix (Thermo Fisher Scientific), 0.25 *μ*l (0.01 pM) of each primer, and 9.5 *μ*l of SDW. The real-time PCR was then analyzed in triplicate. Mean Ct values were used to study the expression of the BCR-ABL region.

### 2.8. Statistical Analysis

For data analysis, SPSS version 18 was used. Results of CML patients were tested by pairwise (before treatment and after treatment). Results of CML patients were exhibited by mean and standard deviation; however, *P* values were used for investigating significance level at <0.05. Results were also expressed by number and their percentages.

## 3. Results

### 3.1. General Characteristics of CML Patients

In a summary of the general characteristics of 131 CML patients presented in Table 1, it was observed that the percentage of CML male patients was more than that of females with a ratio of (1.2 : 1). CML patients were categorized into four age groups. Results demonstrated that most were in the second age group between 31 and 45 years (50 patients; 38.45%), followed by the first age group 16-30 years (47 patients; 35.87%) as compared to other age groups. The old age group above 60 years was 6.87% in this study. Out of 131 individuals, only 41 (34.35%) CML patients were smokers and 90 (68.70%) were nonsmokers, and only one patient had a family history of CML. Sign and symptoms at the time of diagnosis revealed that disturbed sleeping 53.43%, excessive sleeping 49.61%, anxiety 35.87%, depression 37.40%, hair loss 11.45%, night sweating 40.45%, weight loss 35.87%, weight gain 26.71%, and swelling on the body 56.48% were observed in the CML-affected patients. Liver enlargement was observed in 35.87% whereas splenomegaly was observed in 26.71% of the CML patients.

### 3.2. CBC Analysis of All CML Patients

CBC test was performed on all CML patients.  Table 2 displays a summary of WBC, RBC, hemoglobin, HCT, MCH, MCHC, MCV, and platelets. Normal WBC was observed in 22 (16%) patients; however, 75 (57.25%) patients exhibited a very high level of WBC (>100 × 10^3^/*μ*l). RBC within the normal range (4.5–6.5 × 10^3^/*μ*l) was observed in only 24 (18.32%) patients, whereas a reduced number of RBCs were examined in 107 (81.67%) CML patients. Hemoglobin values within the normal range (13-16.5 mg/dl) were observed in 46 (35.11%) cases; however, 71 (58.01%) CML patients showed reduced values of hemoglobin lesser than 10 mg/dl and were found anemic. Hemoglobin values > 13 mg/dl were observed only in 14 (10.68%) CML patients. Hematocrit within the normal range (40-50%) was observed in 17 (12.97%) individuals, and decreased values of hematocrit (<40%) were observed in a maximum number of 114 (87.02%) CML patients. MCH within a normal range (25-32 pg) was found in 80 (61.06%) CML patients, and the induced number of MCH (>32/pg) was examined in 26 (19.84%) patients. MCHC within a normal range (32-36%) was observed in 80 (61.06%) individuals; however, lesser values of MCHC (<32%) were observed in 25 (19.08%) CML patients. Normal values of MCV (76-96 fl) were found in 81 (61.83%) cases, whereas reduced values of MCV (<76 fl) were observed in 29 (22.13%) while induced values of MCV (>96 fl) were observed in 21 (16.03%) CML patients. PLT within the normal range (<150 × 10^3^/*μ*l) was detected in 77 (58.77%) CML cases, whereas the induced number of PLTs (150–400 × 10^3^/*μ*l) was observed in 40 (30.53%) CML patients.

### 3.3. Cytogenetic Study of CML Patients

Table 3 displays the cytogenetic characteristics of CML patients. Cytogenetic tests of 76 CML patients were performed for the diagnosis of CML and the detection of Ph(+ve) and Ph(−ve) cells. The presence of a Ph-positive chromosome was observed in 71 (96.42%) CML patients whereas 5 (6.57%) patients showed Ph(−ve) chromosome. Standard translocations were observed in 63 (82.89%) CML patients. Complex variant translocations with three-way translocations were observed in 3 (7.89%), four-way translocations were observed in 1 (1.31%), and five-way translocations were observed in 1 (1.31%) CML patient. However, additional chromosomal abnormalities were observed in 3 (3.94%) and additional chromosomal abnormalities with complex variant translocations at the same time in the same patient were identified in 1 (1.31%) CML patient. During diagnosis, 66 (92.42%) patients fell in CP, 4 (5.6%) in AP, and 1 (1.40%) in BP. FISH test of 38 CML patients was performed for diagnosis purpose, in which 33 CML patients display BCR-ABL-positive translocations in 200 and/or 500 nuclei counted cells. However, 5 CML patients showed negative translocation.

**Figure 1:**
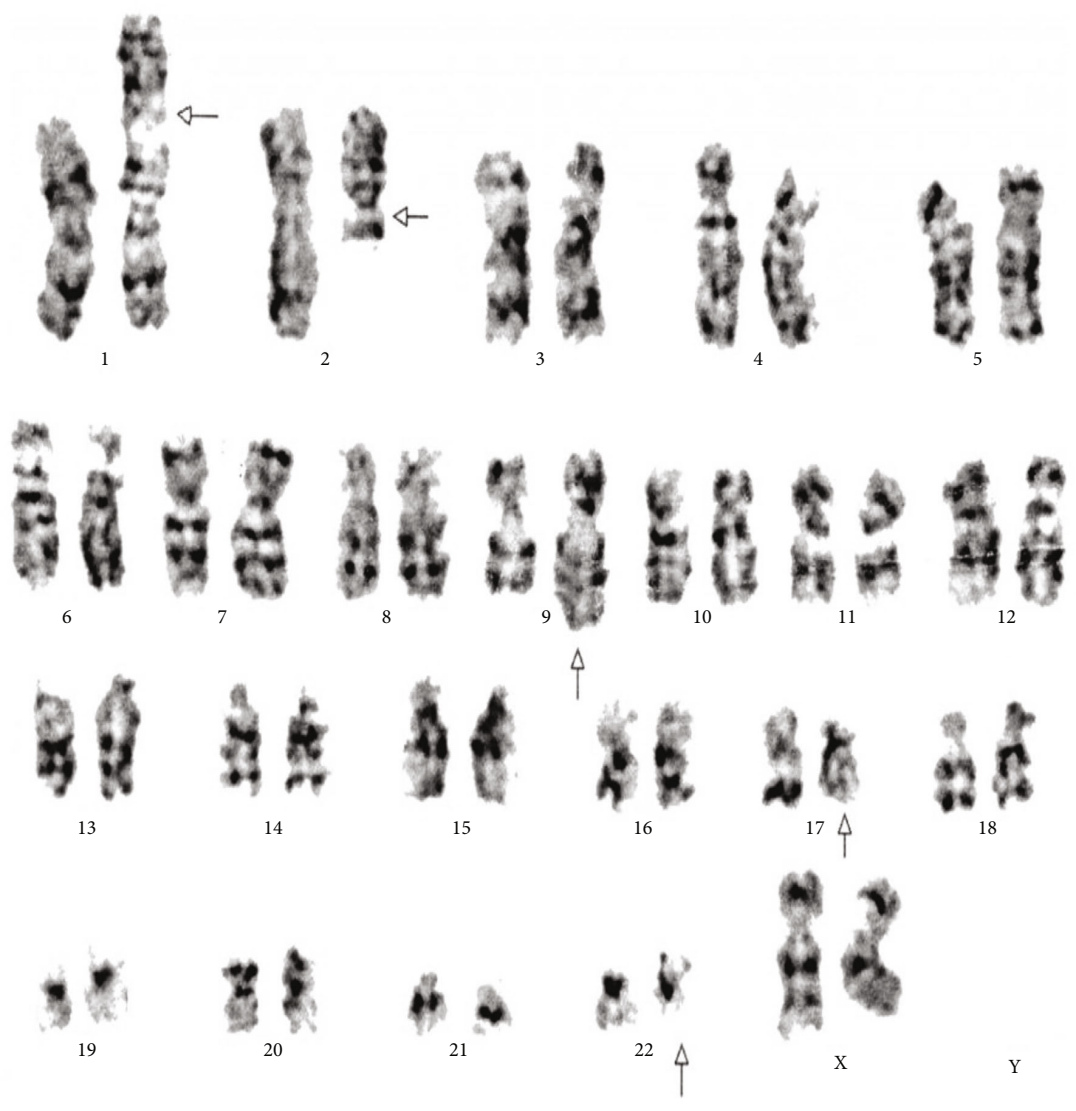
Cytogenetic analysis shows karyotype of five-way complex translocation 46XX,t(1;2;2;17;9;22)(p36.3,q21;q11.2,q21,q34,q11.2) [20].

**Figure 2:**
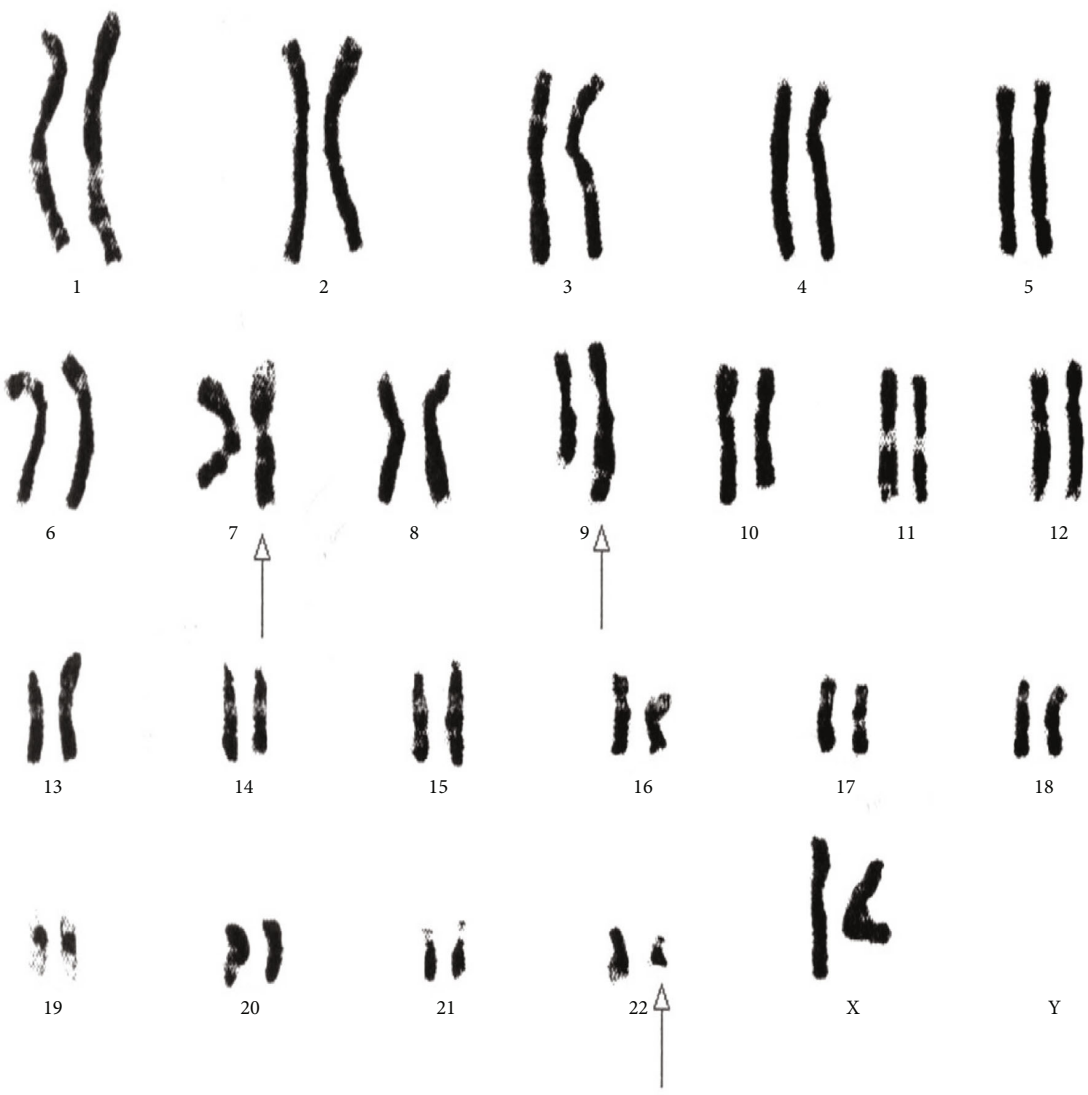
Cytogenetic analysis shows a variant three-way translocation 46XX,t(7;9;22)(p13;q34;q11.2) [20].

**Figure 3:**
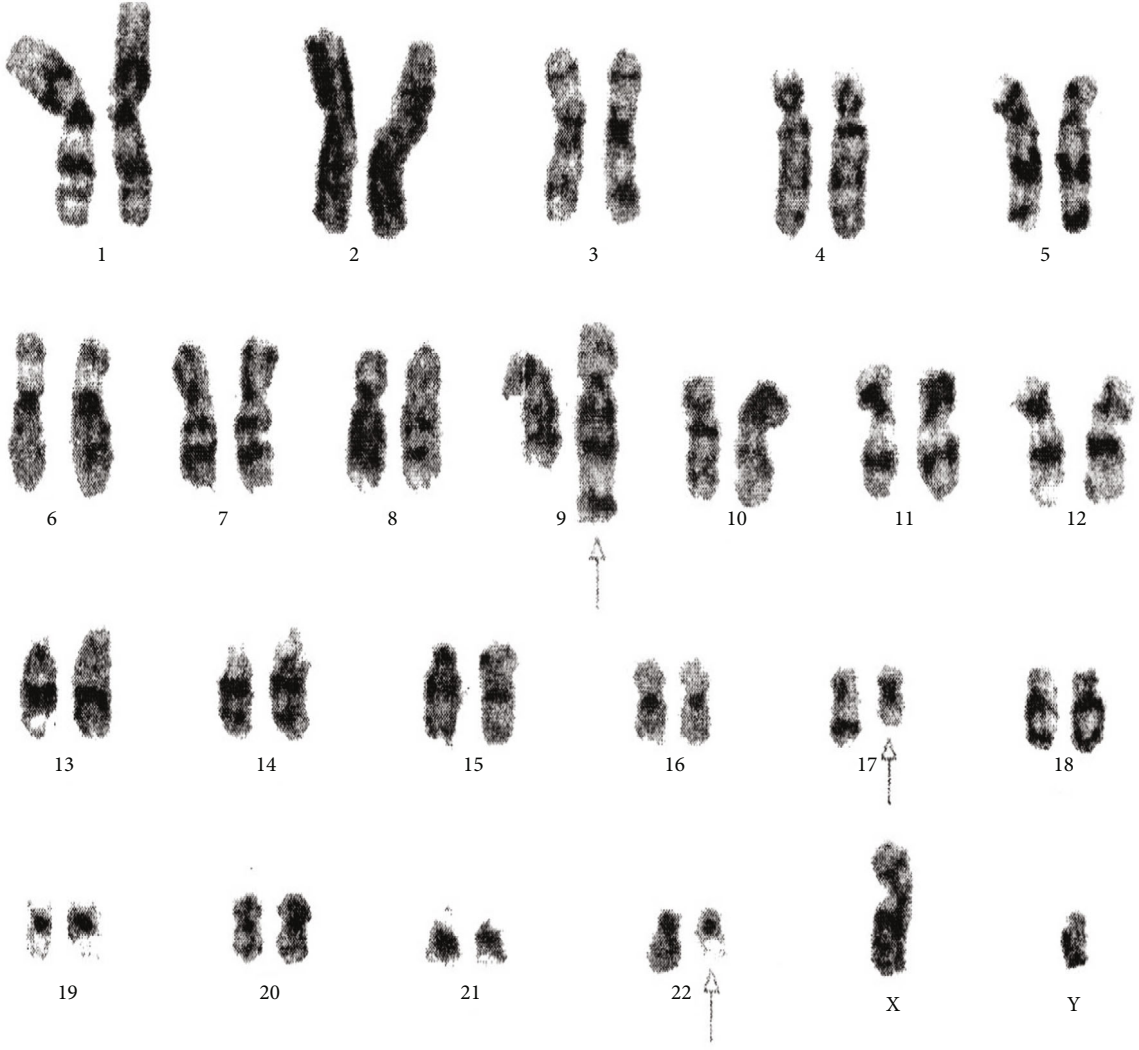
Cytogenetic analysis shows three-way complex variant translocations 46XY,t(9;17;22)(q34;q?11.2;q11.2) [20].

**Figure 4:**
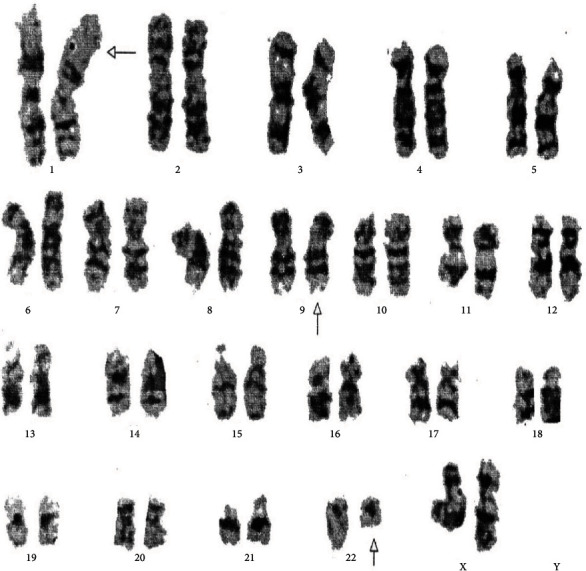
Cytogenetic analysis revealing a variant three-way 46XX,t(1;9;22)(p36.3;q34q11.2) translocation.

**Figure 5:**
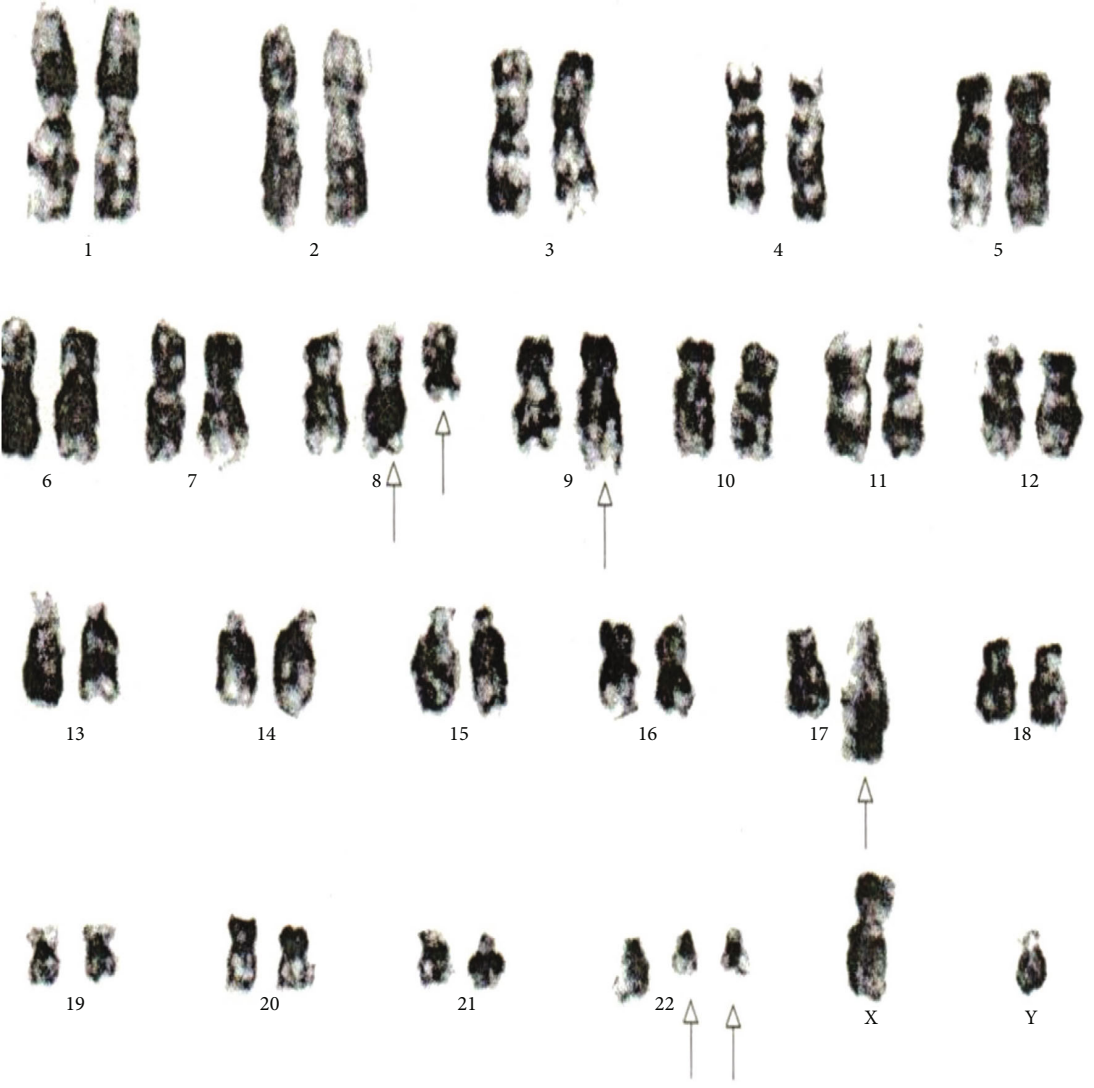
Cytogenetic analysis shows four-way complex/variant translocation, with two additional chromosomes 48XY,+8(8;17),(9;22),+der(22)(q11.2;q23)(q34;q11.2) [20].

**Figure 6:**
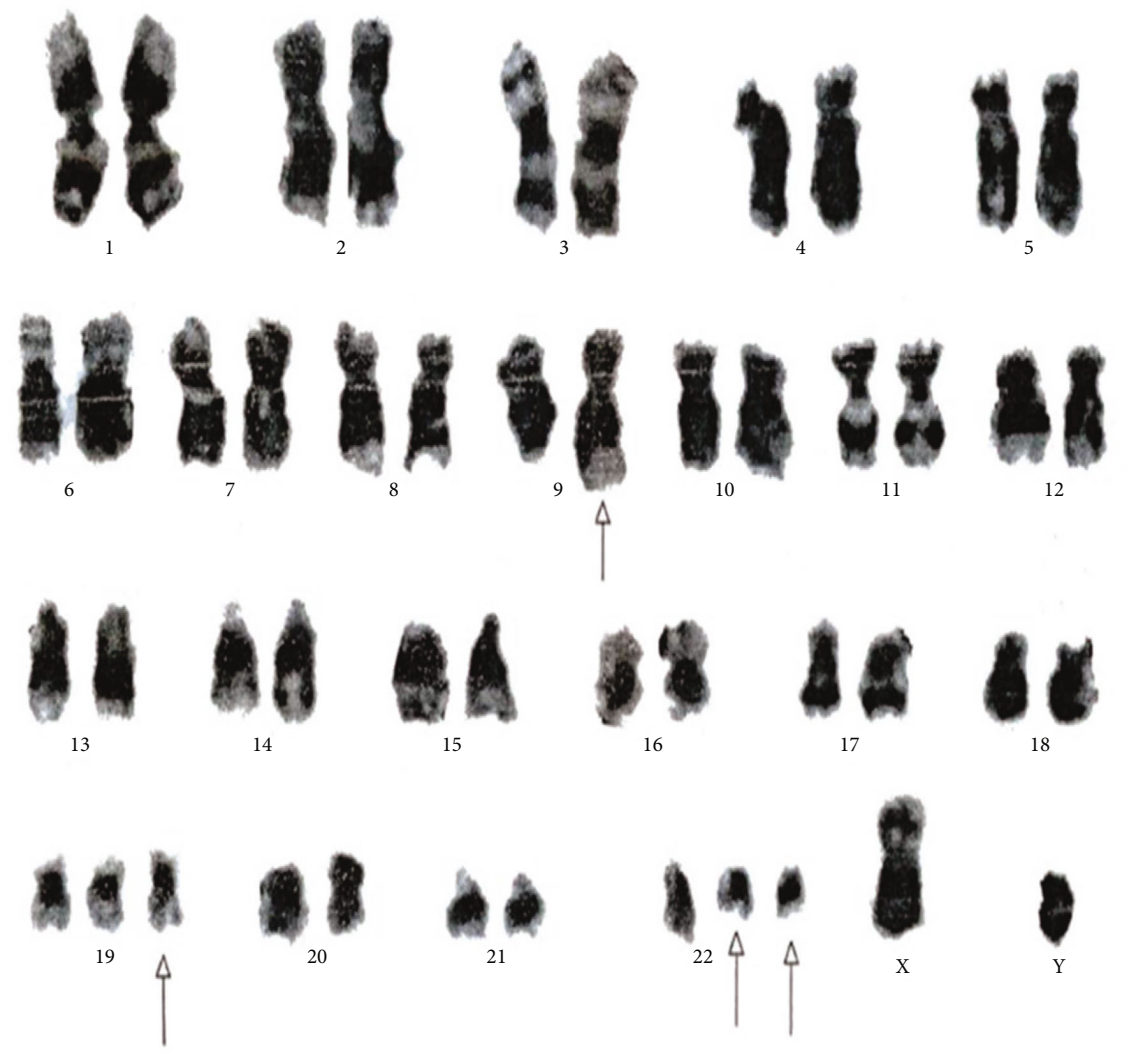
Cytogenetic analysis shows a rare translocation 46XY,t(9;22)(q34;q11.2)[12]/48XY,t(9;22)(q34;q11.2),+19,der(22)t(9;22) [10].

**Figure 7:**
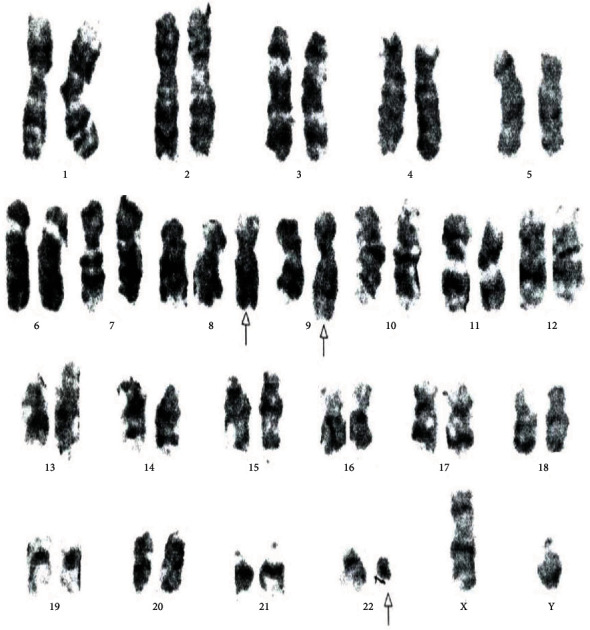
Cytogenetic analysis shows a rare translocation 47XY,+8,t(9;22)(q34;q11.2).

Table 4 displays the complex variant translocations and ACAs. There were a novel five-way translocation 46XX,t(1;2;2;17;9;22)(p36.3,q21;q11.2,q21,q34,q11.2) [20] (the results showed that a segment of chromosome 1p36.3 has been translocated onto chromosome 2q21, the segment of chromosome 2q22 has been translocated onto chromosome 1p36.3 and then breakage of a reunion has occurred between the segments of chromosome 2q11.2 translocation onto chromosome 17q21, and the segment of chromosome 17q21 has been translocated onto chromosome 2q11.2 translocation between chromosomes 9q34 and 22q11.2, resulting in Philadelphia chromosome); a novel four-way translocation with two additional chromosomes 48XY,+8(8;17)(9;22),+der (22)(q11.2;q23)(q34;q11.2) [20] (all cells showed unusual translocation between chromosomes 8q11.2 and 17q23 and another common translocation between 9q34 and 22q11.2, resulting in Philadelphia chromosome with additional chromosomes +8 and an extra derivative chromosome 22); three unique cases of three-way translocations 46XY,t(9;17;22)(q34;q11.2;q11.2), 46XX,t(7;9;22)(p13;q34;q11.2) [20], and 46XX,t(1;9;22)(p36;q34;q11.2) [20]; a unique case of ACAs with Ph-positive chromosome 48XY,t(9;22)(q34;q11.2),+19,der(22)t(9;22) [20]; and two common cases of trisomy 8, 47XY,+8,t(9;22)(q34;q11) [20]. All the important cytogenetic images are shown in the figures (Figures 1–7).

### 3.4. Monitoring and Follow-Up of Hematological Parameters of CML Patients against the Drug Imatinib Mesylate 12-Month Follow-Up

Out of 131 patients, 71 were regularly monitored by CBC after an interval of 6 months. After 12 months of follow-up, WBC, RBC, hemoglobin, PLTs, HCT, lymphocytes, and basophils showed highly significant results (*P* > 0.05) against the drug imatinib mesylate (Table 5).

Table 6 demonstrates that, after 12 months, complete hematologic response (CHR) was observed in 63/71 CML patients. However, only 9 CML patients show increased values of WBC < 10 × 10^3^/*μ*l, decreased values of hemoglobin less than 10 mg/day, and abnormal values of PLTs. In the cytogenetic examination performed on 10 CML patients for monitoring, only 1 showed Ph(+ve) translocation while 9 showed Ph(−ve) translocation complete cytogenetic response. However, 39 CML patients were monitored by FISH between the periods of 6 and 12 months after starting the treatment. After 6 to 12 months of follow-up, 20 CML patients showed the complete response with 0% BCR-ABL gene fusion against the drug. The major response which is the presence of BCR-ABL fusion cells less than 15% was observed in 7 patients, and minor response which is the presence BCR-ABL cells (16-50%) was observed in 4 patients, while no response against the drug which is the presence of BCR-ABL fusion genes (60-100%) was detected in 8 CML patients.

Out of 131 CML patients, 101 patients were successfully treated with imatinib mesylate in which 90 were treated with 400 mg/day and 11 were treated with 600 mg/day. However, 6 CML patients died. The death of 2 CML patients happened after the diagnosis of disease CML within 4 months in the accelerated phase, in which 1 patient died due to drug resistance of imatinib mesylate 600 mg/day while the second died before shifting to any type of drug. However, 4 patients died due to unknown reasons. Moreover, 3 patients died in the chronic phase while one died in the blast phase.

### 3.5. Second-Generation Drug Nilotinib

Out of 131 CML patients, 25 showed resistance against the drug imatinib mesylate and were shifted on second-generation drug nilotinib. Out of the 25 drug-resistant CML patients, 20 were treated with 300 mg/day and 5 were treated with 400 mg/day. Of the 25 patients, 22 were monitored after three months by CBC reverse transcriptase nested PCR and quantitative real-time PCR.

### 3.6. Nested RT-PCR Analysis

**Figure 8:**
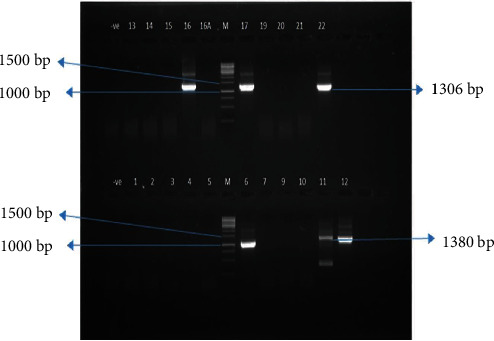
Gel photograph of nested PCR (2nd round) amplification of patients with imatinib resistance mutants. Lane M = 1 kb DNA ladder (Thermo™ SM#0323). Lane 6, 11, 12, 16, 17, and 22 = IM-resistant patients.

Nested PCR was performed for the detection of the BCR-ABL amplification in IM-resistant CML patients. The BCR-ABL gene was successfully amplified in six samples by nested PCR. CML patients have two common variants of fusion oncogene, 1306 bp (b2a2) and 1380 bp (b3a2). Positive amplification was observed in patients numbered 6, 12, 16, 17, and 22 which showed an amplification size of 1306 bp (b2a2) and only 1 showed 1380 bp (b3a2) size (Figure 8).

### 3.7. qRT-PCR Analysis

**Figure 9:**
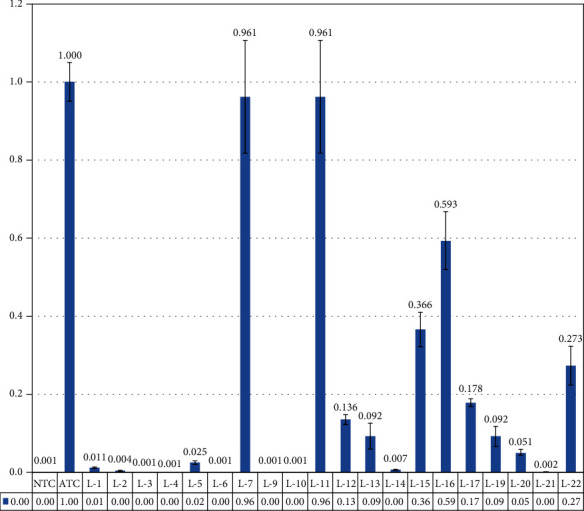
qRT-PCR analysis of imatinib-resistant patient's response against the 2nd-generation. L2, L3, L4, L6, L9, L10, L14, and L21 showed complete molecular response. However, CML patients L1, L5, L13, L19, and L20 showed major molecular response (MMR) BCRABL transcript less than 0.1%. But CML patients L7, L11, L12, L13, L15, L16, L17, and L22 showed no response.

qRT-PCR results shows that of 25/21 after shifting to second-generation drug, 8 patients numbered L2, L3, L4, L6, L9, L10, L14, and L21 showed complete molecular response with 0 percent detection of BCR-ABL cells. However, five CML patients (L1, L5, L13, L19, and L20) showed major molecular response (MMR) reduction of BCR-ABL transcripts less than 0.1%, but CML patients L7, L11, L12, L13, L15, L16, L17, and L22 showed no response against the drug (Figure 9).

## 4. Discussion

The incident CML can appear at any age group; previous studies have shown that it is rare in children. CML is usually diagnosed in the 30-40 age group in the Asian region [35]. Here, we report median age of 38.5 years between the ages of 17 and 75 years. However, the male to female ratio was 1.2 : 1. Aguayo-Gonzalez and Tuna-Aguilar described that the presentation of median age of CML patients was 37 years [36], whereas Faderl et al. stated that the median age presentation of CML patients was between the 45 and 55 years' age group [6]. A study on CML was conducted in Rawalpindi, Pakistan, for a period of 18 months from June 2006 to December 2007 by Ahmed et al. who reported that, out of 45 CML cases, 31 (69%) were men and 14 (31%) were women, whereas men and women ratio was 2.2 : 1 with a mean age of 37.87 years (between the age 18 and 65 years) [37].

CML is associated with pluripotent stem cells. It is a myeloproliferative disorder that includes 15% of adult leukemia. CML prevalence occurs at about 1-2 per one million people in males while 0.6 per one million populations in females [38]. The median age of chronic myeloid leukemia patients is 45-55 years, and most (85%) cases are diagnosed in the chronic phase [8].

The possible symptoms of CML patients are fatigue, abdominal fullness, purpura, splenomegaly, hepatomegaly, bleeding, leukocytosis, anemia, thrombocytosis, anorexia, and weight loss; however, nearly 40% of patients are asymptomatic, and in such cases, the investigation is based exclusively on an abnormal CBC analysis. In these cases, reduced values of hemoglobin and elevated values of white blood cells generally greater than 25,000/mm^3^ and elevated platelet count are observed in 30-40% CML cases. The maximum general aberration on the basis of the physical investigation is splenomegaly, which is present in almost 50% of CML patients [39]. In this current study, symptoms like sleep disturbance 70 (53.47%), excessive sleep 65 (49.61%), night sweating 53 (35.87%), depression 49 (37.40%), splenomegaly 47 (35.87%), hepatomegaly 35 (26.71%), anxiety 47 (82%), swelling on different parts of the body 74 (56.48%), and occasional hair loss 15 (11.45%) are expressively detected in CML patients.

There is very less evidence associating hereditary factors to CML [40]. However, Jaiswal et al. revealed in different studies that hereditary causes and genetic circumstances may increase or suppress the physiological effects of the BCR-ABL gene [38]. We observed in the current study that only 1 patient had a family history of CML.

In the current study, the CBC test of all 131 patients was performed during diagnosis. Upon analysis, we observed that only 22 (16.79%) CML patients fell in the normal WBC range of 11 × 10^3^/*μ*l, 23 (17.55%) CML patients showed induced values of WBC between 11 and 50 × 10^3^/*μ*l, and maximum number of CML patients (75, 57.25%) presented very high values of WBC greater than 100 × 10^3^/*μ*l. In this study, normal PLTs were observed between 150 and 400 × 10^3^/*μ*l in 77 (58.77%) CML patients, while induced values of PLT were found in 40 (30.53%) patients. On the other hand, only 14 (10.68%) CML patients showed a low level of PLT below 150 × 10^3^/*μ*l. Anemic CML patients were investigated which were 117 (89.31%) out of 131; however, only 14 (10.46%) showed a normal range of hemoglobin. Quintas-Cardama and Cortes (2006) reported that laboratory test findings in CML consist of leukocytosis eosinophilia and/or basophilia. PLTs might be either high or low with a normal range of 150–4000 × 10^3^/*μ*l, and mild anemia is frequently observed in all CML patients [14].

Diagnosis of CML mostly depends on cytogenetic and molecular analysis for the identification of the t(9;22)(q34.1;q11.21) Philadelphia chromosome generally counted in 20-25 metaphase cells from bone marrow specimen. The Ph chromosome outcomes from the balanced translocation chromosome of 9 at band q34.1 and 22 at band q11.21.5 [14].

In this study, standard translocation (9;22)(q34.1;q11.21) was observed in 63 (82.89%), complex variant translocations were observed in 4 (5.26%), and additional chromosomal abnormalities were observed in 3 (3.94%), while additional chromosomal abnormalities together with complex variant translocation were observed in 1 (1.31%) CML patient. In this study, 61 (92.42%) CML patients were diagnosed in the chronic phase, 4 (6.06%) were in the accelerated phase, and only 1 (1.51%) CML patient was in the blast phase.

In the current study, we report a novel case of CML patient who exhibited a five-way Ph-positive translocation involving chromosome 46XX,t(1;2;2;17;9;22)(p36.3,q21q11.2,q21,q34,q11.2). To the best of our knowledge, this translocation has not been reported in the literature. Vaidya et al. described that the five-way translocation is a very rare incident in CML [41]. The five-way translocations are very rare in CML patients, and only 13 cases have been reported so far (Table 7) [41–44].

Here, we also report 3 cases of three-way complex translocations. The cases 46XX,t(7;9;22)(p13;q34;q11.2) [20] and 46XY,t(9;17;22)(q34;q?11.2;q11.2) [20] mentioned at Table 4 on serial nos. 3 and 4, to the best of our knowledge, were previously published only once [45, 46]. However, the translocation 46XX,t(1;9;22)(p36.3;q34q11.2) [20] was previously reported seven times (Asif et al., 2015).

Mohamed et al. conducted a study on 58 CML patients. They detected standard Ph-positive translocation in 50 patients and complex/variant translocation involving chromosomes 9 and 22 and an additional third chromosome [47]. Another study was conducted by Fabarius et al. on 1151 CML patients. They examined the standard (9;22) had developed in 1003 (87%) patients, and on the other hand, complex/variant translocations were established in only 69 (6%) CML patients; however, 79 (6.9%) showed ACAs [48].

In this study, we report a unique Ph+ chromosome case in the accelerated phase with additional chromosome of trisomy +19 and +22 der chromosome, 48XY,t(9;22)(q34;q11.2),+19,+der(22)[22]; previously, only one case before has been reported so far [49].

We also investigated another novel case of additional chromosomal abnormalities with four-way complex variant translocation 48XY,+8(8;17)(9;22),+der(22)(q11.2;q23)(q34;q11.2). To the best of our information, this translocation has previously not been reported. So far, only 11 cases of four-way translocations with the involvement of two additional chromosomes were reported in the literature (Table 8).

Al-Achkar (2010) conducted a study on CML that is the same with our finding, a new case of complex variant translocation with additional chromosomal abnormalities 48,XY,+8t(9;22)(q34;q11),der(16),t(16;17),+der(22); the patient failed against the drug imatinib mesylate. During CML evolution, +8 trisomy and extra copy of 22 chromosomes are common anomalies in CML [50].

Wafa et al. defined that additional genetic modifications occur in fewer than 10% of CML cases during diagnosis and other extra genetic changes are obvious in 60-80 percent of CML cases in advanced phases of the disease like AP and BC. The maximum secondary chromosomal anomalies in addition to the Ph(+) chromosome are +8, +Ph, i(17q), and +19. However, minor percentage of CML patients showed chromosomal aberrations like -Y, +21, +17, −7, and −17 (7, 19). Additional cytogenetic abnormalities such as trisomy 8 and Philadelphia chromosome with additional copy are very common in CML cases [51, 52].

In this study, two cases of Philadelphia positive cases with trisomy 8, 47XY,+8,t(9;22)(q34;q11.2) have been reported that are most common in CML. Of the two patients having trisomy +8, one died due to drug resistance; however, the second survived and was successfully treated with IM 400 mg/day.

A study conducted by Wang et al. described trisomy with +8 developed in 37 patients as the sole ACAs. Of the 37 CML patients, 6 showed trisomy +8 at the time of diagnosis; however, trisomy +8 developed during therapy in 31 CML patients. Among these 31 CML patients with extra copy 8 developing for the duration of treatment, on the other hand, 28 CML patients showed +8 in the accelerated phase, while trisomy +8 developed in only 3 CML patients in blast crisis [53].

IM effectively inhibits the tyrosine kinase activity of the BCR-ABL protein and PLT-derived growth factor receptor (PDGFR) which deactivates downstream signaling of the tyrosine kinase through the active site, stumbling cell proliferation and also prompting the process of apoptosis [54].

In this study, out of the 131 CML patients, 71 were initially monitored by physical examination and complete blood count. The CBC test was done twice a year. After 12 months, complete hematologic response (CHR) was observed in 63/71 CML patients. However, only 9 CML patients showed increased values of WBC < 10 × 103/*μ*l, decreased values of hemoglobin less than 10 mg/dl, and abnormal values of PLTs.

Tebuka et al. reported that the achievement of complete hematological response was observed among 90% of patients to IM within three months of treatment. CHR is characterized by a WBC < 10, 000/×10^3^/*μ*l with no juvenile granulocytes and <5% basophils on the differential, and PLTs were less than 450,000/*μ*l. Failing to accomplish a total hematologic response within three months is a sign of an alteration in treatment. Patients with resistance/lack of response against the first-generation drug IM needed to shift to second-generation tyrosine kinase inhibitor therapy. Of the 127 patients, 116 achieved complete hematological response against IM treatment in the first three-month period; however, 11 did not achieve any complete hematological response [55].

In limited CML cases, patients do not display any progress at all or the disease relapse after an initial response. Nearly 60% of patients in BC respond to IM; however, majority of them relapsed. The threat of relapse rises as the disease develops to the next level [56]. On the other hand, resistance to IM can be anticipated by the absence of CHR in CP of CML patients or by the absence of coming back to CP for patients in AP or in BC of CML [57].

Of the 131 patients, 25 CML patients showed resistance against IM, the first-line drug against the CML disease, and shifted on 2nd-generation drug nilotinib in which 22 were analyzed by nested PCR and quantitative real-time PCR. After a median of 3-month follow-up, only 6 CML patients showed BCR-ABL-positive amplification by nested PCR. Of these, 5 showed amplification of oncogene b2a2 on 1306 bp and 1 showed b3a2 on 1380 bp.

Paz-y-Miño et al. researched on 40 CML patients. They observed that frequency of b3a2 transcripts was found in 5.4% CML patients; however, 94.6% showed the b2a2 transcripts [58]. Cabral et al. arranged a study on 97 CML patients. They revealed that b3a2 BCR-ABL transcripts were found in 28% CML patients. On the other hand, 59% of CML patients exhibited b2a2 transcript and 13% showed both b3a2/b2a2 transcripts at the same time [59].

Lemos et al. conducted a study in Brazil executed by RT-PCR for the detection of BCR-ABL on 22 CML patients, in which 59% of the patients showed b3a2 and 41% showed b2a2 transcripts. These previous findings are close to the outcomes of the present study [60]. Another study was conducted by Ghavamzadeh et al. (2008) on 75 CML patients in Iran. They observed that 83% of CML patients showed one of the p210 BCR-ABL transcripts {b3a2 (63%) and b2a2 (20%)} [61].

A study was conducted by Cortes et al. (2007) on 32 CML patients and treated with nilotinib 400 mg/day. The median age of CML patients was 47 years (between ages 24 and 73). The median qRT-PCR follow-up with nilotinib at 3, 6, and 12 months was 0.52%, 0.03%, and 0.09%, respectively. After three-months of follow-up, MMR was detected in 3 out of 22 CML patients (14%), 7 out of 13 (54%) after 6 months, and 5 out of 11 (45%) after 12 months. None of the molecular responses had been lost while on therapy. Physicians reduced the dose of 7 to 400 mg/day and 2 to 200 mg/day, due to extramedullary toxicity, while 3 patients decided to change therapy after 4, 6, and 8 months, respectively, and 2 switched to IM [62]. Another study on 51 CML patients was conducted by Cortes et al. (2010), and in that, 50 (98%) patients achieved a complete response to therapy. However, MMR had been done in 32 (63%) patients, while a complete molecular response was achieved in 12 (24%) CML patients [63].

## 5. Conclusion

In conclusion, here we report a novel five-way complex translocation case 46XX,t(1;2;2;17;9;22)(p36.3,q21;q11.2,q21,q34,q11.2) and a novel case of four-way translocation with two additional chromosomes 48XY,+8(8;17)(9;22),+der(22)(q11.2;q23)(q34;q11.2) in CML patients. Moreover, we also report rare three cases of three-way complex/variant translocations in CML patients that are reported only once so far. In the future, more studies with a larger group of CML patients are required to establish the conclusive effectiveness of current therapies. Next-generation sequencing and animal and cell model studies are needed for the novel and rare translocation cases to further study the effect of these translocations in CML patients.

## Figures and Tables

**Table 1:** General characteristics of CML patients (*n* = 131).

Characteristics	Number	Percentage (%)
Gender distribution (*n* = 131)		
Male	72	54.96
Female	59	45.03
Age group		
16-30	47	35.87
31-45	50	38.16
46-60	25	19.08
Above 60	9	6.87
Smoking status		
Smokers	41	31.29
Nonsmokers	90	68.70
Family history		
Yes	1	0.76
No	130	99.23
Sign and symptoms		
Seeping disturbance	70	53.43
Excessive sleeping	65	49.61
Anxiety	47	35.87
Depression	49	37.40
Hair loss	15	11.45
Night sweating	53	40.45
Weight loss	47	35.87
Weight gain	35	26.71
Swelling on body	74	56.48
Spleen enlargement	35	26.71
Liver enlargement	47	35.87

**Table 2:** Distribution of WBC, RBC, hemoglobin, HCT, MCH, MCHC, MCV, and platelets.

Variables	Number (*N* = 131)	Percentage (%)
WBC normal range 4–11 (10^3^/*μ*l)		
<11 × 10^3^/*μ*l	22	16.79
11.1–50 × 10^3^/*μ*l	23	17.55
50.1–100 × 10^3^/*μ*l	11	8.39
>100 × 10^3^/*μ*l	75	57.25
RBC normal range 4.5–6.5 (10^3^/*μ*l)		
<4.5 × 10^3^/*μ*l	107	81.67
4.6–6.5 × 10^3^/*μ*l	24	18.32
Hemoglobin normal range 13–16.5 (mg/dl)		
<10 mg/dl	71	58.01
10.1–13 mg/dl	46	35.11
>13 mg/dl	14	10.68
Hematocrit normal range 40–50 (%)		
<40	114	87.02
40–50	17	12.97
MCH normal range 25–32 (pg)		
<25 pg	25	19.08
25–32 pg	80	61.06
>32 pg	26	19.84
MCHC normal range 32–36 (%)		
<32%	35	26.71
32–36%	80	61.06
>36%	16	12.21
MCV normal range 76–96 (fl)		
<76 (fl)	29	22.13
76–96 (fl)	81	61.83
≥96 (fl)	21	16.03
Platelet normal range 150–400 (10^3^/*μ*l)		
<150 × 10^3^/*μ*l	14	10.68
150–400 × 10^3^/*μ*l	77	58.77
>400 × 10^3^/*μ*l	40	30.53

**Table 3:** Cytogenetic results of CML patients, phases at the time of diagnosis (*N* = 71), and FISH analysis (*N* = 38).

Cytogenetic test		Percentage (%)
Ph (+ve) (9;22)(q34;q11)	71	93.42
Ph (−ve) (9;22)(q34;q11)	5	6.63
Standard translocations	63	82.89
Variant translocation complex		
Three-way translocations	3	7.89
Five-way translocations	1	1.31
Additional chromosomal abnormalities	3	3.94
Complex variant translocations together with additional chromosomal abnormalities	1	1.31
Phase of CML disease		
Chronic phase	66	92.95
Accelerated phase	4	5.6
Blast phase	1	1.40
FISH analysis at diagnostic stage		
FISH (+)	33	86.84
FISH (−)	5	13.15

**Table 4:** Cytogenetic characteristics features of CML patients exhibit additional chromosomal abnormalities and complex variant translocations.

S. no	Translocations	Age	Sex	CBC								Treatment		Phase	Major Sign & Symptoms	Figure #
				WBC ×103/ul	RBC ×103/ul	Hb g/dl,	PLT ×103/ul	MCV/fl,	MCH/pg	HCT %	MCHC g/dl	Imatinib Mesylate mg/day	Nilotinib mg/day			
1	46XX,t(1; 2; 2; 17; 9; 22)(p36.3,q21; q11.2,q21,q34,q11.2	40	Female	122	4.1	10.7	404	80	26	34	31		300	Chronic	Anemia, weight loss, fever	Figure 1
2	46XX,t(7; 9; 22)(p13; q34; q11.2)	31	Female	533	2.51	8.1	364	85	32.3	22	37.7	400		Chronic	Anemia, sweating, fever, weight loss	Figure 2
3	46XX,t(9; 17; 22)(q34; q?11.2; q11.2)	27	Male	44.3	3.23	11.3	426	95.8	32	33.8	33.4	400		Chronic	Anemia, weakness, fever, swelling	Figure 3
4	46XX, t (1; 9; 22) (p36.3; q34; q11.2)	45	Female	379	2.44	8.2	449	88.5	22.2	27.6	35.6	400		Chronic	Anemia, weight loss, fever, splenomegaly	Figure 4
5	48XY,+8(8; 17)(9; 22),+der(22)(q11.2; q23)(q34; q11.2)	32	Male	89.6	2.9	6.1	25	105	27.5	23.3	30	600		Accelerated	Weight loss, bone pains, Anemia	Figure 5
6	48XY,t(9; 22)(q34; q11.2),+19,der(22)t(9; 22)[20]	75	Male	19.4	2.69	10.4	295	81		75.5	32,7	600		Accelerated	Fever, weight loss, anxiety, depression	Figure 6
7	47XY, +8, t (9; 22) (q34; q11). [20]	55	Male	188.2	2.6	8.5	613	97.3	32.4	27.7	33.4	600		Accelerated	Anxiety, sleep disturbance sweating, Anemia,	Figure 7
8	47XY,+8,t(9; 22)(q34; q11) [20]	46	Male	16	3.83	10	26.1	82.5	26.1	27.7	31.6			Chronic	Weight loss, fever, depression	—

**Table 5:** Hematological parameter analysis by paired *t*-test of CML patients for monitoring before and after treatment of 12 months against imatinib mesylate.

Variables	Paired differences			*T*	df	Sig.
	Mean	Std. deviation	Std. error mean			
WBC	178.88127	163.32559	19.38318	9.229	70	**0.000**
Hbg	-1.68704	2.66257	0.31599	-5.339	70	**0.000**
PLT	74.57746	213.00172	25.27865	2.950	70	**0.004**
RBC	-0.64732	1.04391	0.12389	-5.225	70	**0.000**
MCH	0.57366	5.75767	0.68331	0.840	70	0.404
MCHC	1.22380	6.65783	0.79014	1.549	70	0.126
HCT	-5.85056	9.87587	1.17205	-4.992	70	**0.000**
MCV	-1.08873	15.97131	1.89545	-0.574	70	0.568
Lym	-15.22113	19.54470	2.31953	-6.562	70	**0.000**
Mono	-0.61127	3.11290	0.36943	-1.655	70	0.102
NEO	-3.74859	19.69170	2.33697	-1.604	70	0.113
Eos	1.49549	5.08325	0.60327	2.479	70	**0.016**
Baso	0.91831	2.44238	0.28986	3.168	70	**0.002**

**Table 6:** Results of CML patients who responded to imatinib mesylate treatment.

Response	After 6 months	After 12 months
Complete hematologic response (*n* = 71)	60 (84.50%)	63 (88.73%)
Partial hematologic response	3 (4.22%)	4 (5.63%)
No response	8 (11.26%)	4 (5.63%)
Cytogenetic (Ph) response (*n* = 10)		
Complete	9 (90%)	—
No response	1 (10%)	—
FISH (BCR-ABL) (*n* = 39)		
Complete (BCR-ABL 0%)	17 (43.58%)	3 (7.69%)
Partial (BCR-ABL 1–15%)	6 (15.38%)	1 (2.56)
Minor (BCR-ABL 35–50)	4 (10.25%)	—
No response (BCR-ABL 50–100%)	6 (15.38%)	2 (5.12%)

**Table 7:** Previously reported five-way Ph complex variant translocations in literature.

No.	Translocations reported in previous literature	Reference
1	46,XY,t(4;18;13;9;22)(q12;q11;q14;q34;q11)	[43]
2	46,XY,t(4;18;13;9;22)(q12;q11;q14;q34;q11)	[64]
3.	46,XY,t(9;10;15;19;22)(q34;q22;q22;q13;q11)	[65]
4	t(3;4;9;11;22)(p2l;q34;ql3;ql1)	[66]
5	46XX,t(9;22;21;11;inv ins(12))(q15p12p13)(q34;q11;q22;q13;q1)	[67]
6	46XY,t(9;22;10;12;1)(q34;q11.2;q22;p12;p36.1)	[68]
7	46XX,t(9;22;15;13;17)(q34;q11;q26;q14;q11)	[69]
8	46XY,t(2;9;16;22;22)(q32;q34;q21;q11;q11)	[46]
9	46XY,t(4;12;7;9;22)(q33?;q24;p13;q34;q11)	[70]
10	46XX,t(1;4;5;9;22)(q42;p14;q31;q34;q11.2)	[71]
11	46XY,t(7;11;9;22;9)(q22;q13;q34;q11.2;q34)	[42]
12	46,XY,t(6;10;9;9;22)(q24;p15;p13;q34;q11)	[44]
13	46,XY,t(9;11;13;19;22)(9q34.12;11p11.12;13q21.31;19q13.12;22q11.21)	[41]
14	46XX,t(1;2;2;17;9;22)(p36.3,q21;q11.2,q21,q34,q11.2)	This study

**Table 8:** Previous reported four-way translocations with two additional chromosomes.

S. no.	Translocations reported in previous literature	Reference
1	48,XY,t(3;21)(q26;q22),+8,t(9;22)(q34;q11),+der(22)t(9;22)	[72]
2	48,XX,t(9;22)(q34;q11),t(13;14)(q14;q24-32),+19,+der(22)t(9;22)	[73]
3	48,XY,+8,t(9;22),der(10)t(1;10)(q11;p12)	[74]
4	48,XY,t(3;14)(q26;q11),t(9;22)(q34;q11),+der(22)t(9;22)x2	[75]
5	48,XY,+Y,t(7;22)(p22;q11),+8,t(9;22)(q34;q11),i(17)(q10)	[76]
6	48,XX,t(7;9)(q35;p13),t(9;22)(q34;q11),t(12;13)(q24;q22),+2mar	[77]
7	48,XY,+8,t(9;15),t(9;22),+13,-15,+i(22)(q10)	[78]
8	48,XX,t(3;3)(q21;q29),+8,t(9;22)(q34;q11),+16	[79]
9	48,XY,t(7;11),+8,t(9;22),+der(22)t(9;22)	[80]
10	48,XY,t(8;22),t(9;22)(q34;q11),+14,+20	[81]
11	48,XX,der(7)t(2;7)(q21;p15)del(2)(q35),t(9;22)(q34;q11),+13,+mar	[82]
12	48XY,+8(8;17)(9;22),+der(22)(q11.2;q23)(q34; q11.2)	This study

## Data Availability

Data is fully available upon request and stored in Department of Biotechnology, BUITEMS.
